# Case Study: The use of drugs in the management of behavioral and psychological symptoms in Dementia

**DOI:** 10.1002/alz70859_102310

**Published:** 2025-12-25

**Authors:** Juliana de Oliveira Alves, Marcelo Henrique Quaresma Castro Salomão

**Affiliations:** ^1^ Universidade Feevale, Novo Hamburgo, Rio Grande do Sul Brazil; ^2^ Pontifícia Universidade Católica de Campinas, São Paulo, São Paulo Brazil

## Abstract

**Background:**

Dementia affects approximately 47 million people globally. Behavioral and psychological symptoms of dementia (BPSD), such as agitation, aggression, delusions, and hallucinations, are common during the progression of the disease and directly impact the quality of life of patients, families, and caregivers (CALSOLARO, 2021; FRANCHI, 2016). This study analyzes pharmacological management strategies for these cases.

**Method:**

A comparative analysis was conducted using three scientific articles from the Medline database that address pharmacological treatment in patients with dementia.

**Result:**

Antipsychotics are frequently used to treat agitation, aggression, and psychosis in dementia. Typical antipsychotics: Haloperidol is widely used for delirium but presents a high risk of side effects, such as sedation, extrapyramidal symptoms, and increased mortality, being recommended only in emergencies (CALSOLARO, 2021). Atypical antipsychotics: These are more tolerable, with a lower incidence of extrapyramidal symptoms. Risperidone is effective in managing agitation and aggression (FRANCHI, 2016) and is indicated for moderate to severe Alzheimer’s cases (CALSOLARO, 2021). Aripiprazole reduces psychotic symptoms with fewer side effects (MINTZER, 2007), while quetiapine is preferred for patients with Parkinsonian characteristics (CALSOLARO, 2021). However, the prolonged use of atypical antipsychotics is associated with increased mortality and cerebrovascular events (CALSOLARO, 2021). Antidepressants: SSRIs, such as citalopram, may help with agitation but carry the risk of cognitive decline and QT interval prolongation (FRANCHI, 2016). Acetylcholinesterase inhibitors and memantine: Rivastigmine and memantine reduce agitation and aggression; donepezil provides small improvements (FRANCHI, 2016). Analgesics: Paracetamol alleviates pain and reduces neuropsychiatric symptoms, while opioids decrease agitation (FRANCHI, 2016). Anticonvulsants: Carbamazepine showed limited benefits, but its tolerability restricts its use; valproate did not demonstrate efficacy (FRANCHI, 2016).

**Conclusion:**

Pharmacological management should be individualized, considering symptoms, comorbidities, and frailty. Medications should be used at the lowest effective dose and for the shortest possible duration to minimize side effects.

